# Combination of herbivore removal and nitrogen deposition increases upland carbon storage

**DOI:** 10.1111/gcb.12902

**Published:** 2015-04-30

**Authors:** Stuart W. Smith, David Johnson, Samuel L. O. Quin, Kyle Munro, Robin J. Pakeman, René van der Wal, Sarah J. Woodin

**Affiliations:** ^1^ Institute of Biological and Environmental ScienceUniversity of AberdeenSt Machar DriveAberdeenAB24 3UUUK; ^2^ The James Hutton InstituteCraigiebucklerAberdeenAB15 8QHUK; ^3^ ACESUniversity of AberdeenSt Machar DriveAberdeenAB24 3UUUK

**Keywords:** *Calluna vulgaris*, exclosures, grazing, heathlands, *Molinia caerulea*, nitrogen deposition, plant litter, soil carbon

## Abstract

Ecosystem carbon (C) accrual and storage can be enhanced by removing large herbivores as well as by the fertilizing effect of atmospheric nitrogen (N) deposition. These drivers are unlikely to operate independently, yet their combined effect on aboveground and belowground C storage remains largely unexplored. We sampled inside and outside 19 upland grazing exclosures, established for up to 80 years, across an N deposition gradient (5–24 kg N ha^−1^ yr^−1^) and found that herbivore removal increased aboveground plant C stocks, particularly in moss, shrubs and litter. Soil C storage increased with atmospheric N deposition, and this was moderated by the presence or absence of herbivores. In exclosures receiving above 11 kg N ha^−1^ year^−1^, herbivore removal resulted in increased soil C stocks. This effect was typically greater for exclosures dominated by dwarf shrubs (*Calluna vulgaris*) than by grasses (*Molinia caerulea*). The same pattern was observed for ecosystem C storage. We used our data to predict C storage for a scenario of removing all large herbivores from UK heathlands. Predictions were made considering herbivore removal only (ignoring N deposition) and the combined effects of herbivore removal and current N deposition rates. Predictions including N deposition resulted in a smaller increase in UK heathland C storage than predictions using herbivore removal only. This finding was driven by the fact that the majority of UK heathlands receive low N deposition rates at which herbivore removal has little effect on C storage. Our findings demonstrate the crucial link between herbivory by large mammals and atmospheric N deposition, and this interaction needs to be considered in models of biogeochemical cycling.

## Introduction

Land use management is widely acknowledged as a key controlling factor of C storage in many of the world's ecosystems. However, the effectiveness of land use management will depend on how it interacts with other environmental drivers, such as atmospheric nitrogen (N) deposition, which is a significant source of N for northern ecosystems (Bobbink *et al*., 2010). For example, fertilization by atmospheric N deposition has been shown to enhance ecosystem C storage in deciduous and boreal forests and heathlands across Europe and the United States (Hyvönen *et al*., 2008; De Vries *et al*., 2009). Increasing N availability stimulates plant productivity and litter production, enhancing the accumulation of organic matter (Carroll *et al*., 2003; Currey *et al*., 2010; Tipping *et al*., 2012). Little is known about the interactive effect of N deposition with land management practices which influence C storage, such as herbivore grazing. Excluding herbivores or reducing grazing pressure is considered important strategies for increasing plant and soil C storage in many ecosystems (Piñeiro *et al*., 2010; Tanentzap & Coomes, 2012). Yet, the occurrence and direction of an interactive effect of herbivore exclusion and atmospheric N deposition on C storage remain uncertain. On the one hand, combined effects could increase C storage due to the increase in plant productivity, litter accumulation and plant C inputs to the soil (Hartley, 1997; Van der Wal *et al*., 2003; Emmett *et al*., 2004; Hartley & Mitchell, 2005). On the other hand, combined effects may reduce C storage by reducing the recalcitrance of plant litter (i.e. lower C : N ratio) and/or shifting plant C inputs belowground, thereby mobilizing microbes to decompose stored soil C (Mack *et al*., 2004; Bragazza *et al*., 2006, 2012; Hartley *et al*., 2012).

Upland areas of NW Europe (areas generally >200 m. a.s.l., where farming becomes less profitable due to the limited productivity of the land; Reed *et al*., 2009) are globally important reservoirs of C, and so it is crucial to better understand how herbivore removal and N deposition interact to affect C storage in these systems. Heather (*Calluna vulgaris* (L.) Hull)‐dominated wet upland heathlands have high soil C concentrations (mean 284.9 g C kg^−1^) and densities (mean 8.4 kg C m^−2^) in the top 15 cm of the soil profile, which need to be maintained to ensure long‐term C storage (Emmett *et al*., 2010). The majority of the world's upland heath is found in the UK (1.9 million ha; Carey *et al*., 2008), and it covers a wide gradient in N deposition (Southon *et al*., 2013), thus presenting an ideal system to study the effects of N deposition on C storage. Upland heathlands are nutrient‐limited systems and considered threatened by N deposition with a recommended critical load of 10–20 kg N ha^−1 ^year^−1^ (Bobbink & Hettelingh, 2010). The critical load is defined as the threshold above which some change in a sensitive element of the environment (e.g. lichen or moss species abundance) is predicted to occur according to present knowledge. These systems are also extensively grazed by livestock (sheep and cattle) and deer, which exert greater impact on upland heath and coarse grass vegetation than all other herbivores (Albon *et al*., 2007). Across UK heathlands, there is uncertainty as to the long‐term impact of recent declines in livestock numbers on C storage (Van der Wal *et al*., 2011).

There is growing interest in the impact of herbivore removal, N deposition and the relative abundance of shrub and graminoid species on the C balance of northern ecosystems (see Mack *et al*., 2004; Olofsson *et al*., 2009; Sjögersten *et al*., 2011; Gill, 2014). Net C storage in upland heathlands has been shown to be related to the abundance of the dwarf shrub *C. vulgaris* because this species has more recalcitrant plant litter compared to co‐dominant graminoid species (Ward *et al*., 2007, 2013; Medina‐Roldán *et al*., 2012; Quin *et al*., 2014). Elevated N deposition can result in a loss of *C. vulgaris* and an increase in grass species such as *Molinia caerulea* (L.) Moench in upland heathland (Ross *et al*., 2012; Southon *et al*., 2013). This change in species dominance is not a result of N addition alone, because *C. vulgaris* often remains a superior competitor for light at high N addition rates (Aerts *et al*., 1990; Power *et al*., 1998). Instead, if the *C. vulgaris* canopy is disturbed by herbivore grazing and there is high N availability, then grasses such as *M. caerulea* may take over, because they have a greater growth response to N (Hartley, 1997; Emmett *et al*., 2004; Hartley & Mitchell, 2005).

Changes in C and N cycling in wet upland heathlands are generally slow (i.e. detectable on a decadal timescale), yet the duration of exclosure experiments previously used to investigate these changes has typically been <10 years (Medina‐Roldán *et al*., 2012; Tanentzap & Coomes, 2012; Smith *et al*., 2014a). Such limited exclosure duration reduces the likelihood of detecting significant differences in plant and soil C storage inside and outside exclosures; therefore, such experiments have not provided empirical evidence of an increase in long‐term C pools in soil following the removal of herbivores from wet upland heathlands (Garnett *et al*., 2000; Ward *et al*., 2007; Medina‐Roldán *et al*., 2012). By contrast, increases in soil C pools have been detected following N addition (Hyvönen *et al*., 2008; De Vries *et al*., 2009; Bragazza *et al*., 2012). *C. vulgaris‐*dominated communities have not been studied across a sufficient range of N inputs to enable detection of the potential stimulatory effects of herbivore exclusion and N addition on soil C storage. Utilizing a spatial approach of studying herbivore removal across a ‘natural’ gradient of N deposition (see Stevens *et al*., 2004; Armitage *et al*., 2011) could elucidate the potential interactive effects of these factors on C storage in shrub‐ and grass‐dominated upland heathlands.

In this study, we utilized established grazing exclosures in wet upland heathlands across the northern part of the UK (where most of this habitat is found). We surveyed both aboveground and belowground C stocks inside and outside long‐term exclosures (ages ranging from 5 to 80 years) across a regional gradient of N deposition (5–24 kg N ha^−1 ^yr^−1^). We also accounted for regional variation in long‐term climatic variables that potentially influence plant and soil C stocks. This approach enabled us to address the following questions: (1) Does exclusion of large herbivores (usually sheep) for up to 80 years affect plant and soil C stocks? (2) Does N deposition influence the response of C stocks to exclusion of herbivores, and if so, (3) what impact would herbivore removal from heathlands have on UK C stocks given current spatial patterns and rates of N deposition? Crucially, this study addresses whether greater consideration needs to be given to the potential interdependent effects of grazing management and N deposition on C storage in upland heathlands.

## Materials and methods

### Site selection and field surveying

**Figure 1:**
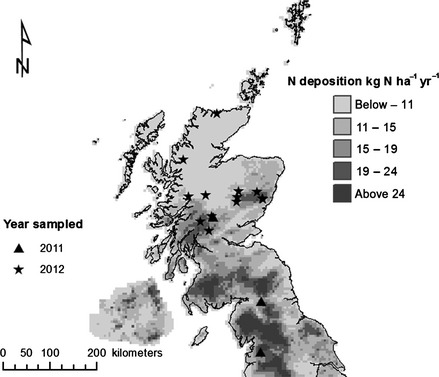
Surveyed exclosure locations across the UK uplands in relation to spatial variation in total atmospheric N deposition (2011; http://pollutantdeposition.defra.gov.uk/pollutant-maps). A total of 15 locations are shown; at three locations, two or three exclosures, approximately 5 km apart, were surveyed (*n* = 19).

Nineteen exclosures across upland areas of the UK were selected with similar characteristics (dominant plant species, major soil types, elevation, slope, aspect) across a gradient of modelled N deposition spanning 5–24 kg N ha^−1 ^year^−1^ (Fig. 1; Table 1; Concentration‐based Estimated Deposition (CBED) model using 5 × 5 km grids accessed via http://www.apis.ac.uk/; Smith *et al*., 2000). Exclosures were selected based on N deposition rates for 2011. While N deposition rates may have changed over the years the exclosures have been in place, the ranking of sites by their rates of N deposition has remained unchanged for over a decade (comparison between 2011 and 1996–1998; Wilcox test; *W* = 866, *P* = 0.14; Table 1). Upland exclosures (averaging 352 m. a.s.l.) were chosen to represent northern wet heathland plant communities dominated by the dwarf shrub *C. vulgaris* or the grass *M. caerulea* (Table 1). These communities were associated with organic soils, including blanket peats, peaty gleys/podzols and humus‐iron podzols, with soil C concentrations ranging from 4 to 50% to a depth of 15 cm (Table 1; www.soils-scotland.gov.uk). One site (Invernaver) differed from the others in that the exclosure was dominated by *Juniperus communis* subsp. *nana* (Hook.) Syme. (Table 1). The site was retained, however, as *J. communis* subsp*. nana* often coexists with *C. vulgaris,* and both species respond similarly to N fertilization (McGowan *et al*., 1998). The exclosures ranged in age from 7 to 80 years (Table 1) and had typically been erected to exclude sheep from vegetation, although the fencing equally prohibited access by red deer (*Cervus elaphus*), cattle and, at many exclosures, rabbits (*Oryctolagus cuniculus*) and mountain hares (*Lepus timidus*) (Table S1).

**Table 1:** Exclosure locations (UK national grid reference), atmospheric nitrogen deposition for 2011 (1996–1998 subscript in parenthesis^a^), exclosure age, altitude, pellet density outside exclosures, dominant plant functional group and species inside and outside exclosures, and soil type and association. Ben Lawers, Bowland and Geltsdale were sampled in 2011 (see Quin *et al*., 2014), and pellet densities were not measured; all other sites were sampled in 2012

Site	National grid reference	N deposition in 2011_(1996/98)_ (kg N ha^−1^ yr^−1^)	Age (years)	Altitude (m)	Pellets (m^−2^)	Functional group (dominant species)		Soil
						Exclosure	Grazed	Type (association)
Ballogie	NO557935	20.6_(17.8)_	7	180	0.02	Shrub (*Calluna vulgaris*)	Shrub (*Calluna vulgaris*)	Freely drained iron podzol (Countesswells)
Beinn Eighe	NG980626	8.0_(9.3)_	53	470	0.2	Shrub (*Calluna vulgaris*)	Shrub (*Calluna vulgaris*)	Peaty podzol (Durnhill)
Ben Lawers	NN611381	12.9_(14.5)_	22	480	–	Shrub (*Calluna vulgaris*)	Grass (*Nardus stricta*)	Humus‐iron podzol (Strichen)
Bowland	SD625502	23.7_(30.8)_	14	280	–	Shrub (*Calluna vulgaris*)	Grass (*Molinia caerulea‐Nardus stricta*)	Poorly drained peat
Creag Meagaidh (plot C)	NN463867	7.3_(7.8)_	25	320	0	Shrub (*Calluna vulgaris*)	Shrub (*Calluna vulgaris*)	Peaty podzol (Kilodian)
Creag Meagaidh (plot D)	NN455859	7.3_(7.8)_	25	360	0.02	Shrub (*Calluna vulgaris*)	Shrub (*Calluna vulgaris*)	Peaty gleys (Badanloch)
Crianlarich	NN350301	16.8_(19.1)_	16	380	0.12	Grass (*Molinia caerulea*)	Grass (*Molinia caerulea*)	Peaty podzol (Strichen)
Geltsdale	NY645580	16.5_(19.2)_	15	240	–	Shrub (*Calluna vulgaris*)	Grass (*Molinia caerulea*)	Poorly drained blanket bog peat
Glen Clunie	NO139820	14.7_(12.9)_	19	450	0.28	Shrub (*Calluna vulgaris*)	Shrub (*Calluna vulgaris*)	Peaty podzol (Strichen)
Glen Finglas (block B)	NN529109	15.3_(20.3)_	9	300	0.03	Grass (*Molinia caerulea*)	Grass (*Molinia caerulea*)	Humus‐iron podzol (Strichen)
Glen Finglas (block C)	NN483122	16.8_(20.5)_	9	460	0.06	Grass (*Molinia caerulea*)	Shrub/grass (*Calluna vulgaris*–*Vaccinium myrtillus–Deschampsia flexuosa*)	Humus‐iron podzol (Strichen)
Glen Finglas (block E)	NN515141	15.3_(20.3)_	9	330	0.04	Shrub (*Calluna vulgaris*)	Grass (*Molinia caerulea*)	Humus‐iron podzol (Strichen)
Glen Loy	NN093837	8.1_(10.4)_	80	280	0.03	Shrub (*Calluna vulgaris*)	Grass (*Molinia caerulea*)	Peaty podzol (Kilodian)
Glen Shee	NO125725	12.9_(13.4)_	19	440	0.06	Shrub (*Calluna vulgaris*)	Shrub (*Calluna vulgaris*)	Humus‐iron podzol (Stirchen)
Glensaugh (MOORCO)	NO675799	17.5_(18.7)_	7	310	0.25	Shrub (*Calluna vulgaris*)	Shrub/grass (*Calluna vulgaris*‐*Vaccinium myrtillus‐Deschampsia flexuosa*)	Peaty podzol (Strichen)
Glensaugh (Strathfinella Hill)	NO677780	17.5_(18.7)_	21	270	0.18	Shrub (*Calluna vulgaris*)	Shrub/grass (*Calluna vulgaris*‐*Vaccinium myrtillus‐Deschampsia flexuosa*)	Freely drained iron podzol (Strathfinella)
Invercauld	NO165946	12.2_(11.7)_	7	520	0.02	Shrub (*Calluna vulgaris*)	Shrub (*Calluna vulgaris*)	Peaty podzol (Arkaig)
Invernaver	NC694616	5.3_(6.9)_	34	60	0.22	Shrub (*Juniperus communis subsp. nana*‐*Salix repens*)	Shrub (*Dryas octopetala*)	Freely drained Calcareous regosol (Fraserburgh)
Loch na Lairigie	NN593412	14_(17.1)_	12	550	0.12	Grass (*Molinia caerulea*)	Grass (*Molinia caerulea*)	Peaty podzols (Garlie)

a 1996–1998 N deposition data provided by Ron Smith, Centre for Ecology & Hydrology, Edinburgh, UK.

Selection of the sampling area within exclosures and adjacent grazed areas was based on vegetation being representative of the area, typically *C. vulgaris* or *M. caerulea*‐dominated communities (Table 1). Both inside and outside exclosures, the final sampling area was selected following random cardinal directions stratified within the representative dominant vegetation type. The sampling area for the grazed vegetation was a maximum distance of 30 m from the exclosure sampling area to minimize variation in microclimate and edaphic conditions. The grazed sampling area was always a minimum distance of 5 m from the fence‐line to avoid sampling vegetation that is intensely disturbed or grazed by herbivores at the exclosure boundary. At sites with multiple exclosures, an individual exclosure was only sampled if it was a minimum distance of 5 km from another sampled exclosure to reduce spatial covariation in environmental variables (e.g. N deposition, rainfall and temperature). In instances when there were multiple exclosures within a 5 km radius, exclosures were selected at random, after excluding any which differed significantly from selection criteria outlined above. At four sites, the exclosures were part of mountain shrubland and woodland restoration projects and contained tree seedlings that were <0.5 m tall (Ben Lawers, Creag Meagaidh exclosures C and D and Loch na Lairige; Table 1). Within these exclosures, it was not possible to differentiate between natural tree regeneration and planted trees. However, total tree seedling densities were low, averaging 0.22 seedlings m^−2^ (ranging from 0.003 to 0.58 trees m^−2^) across only four exclosures, in which plant communities were still dominated by *C. vulgaris* and *M. caerulea* (Mardon, 2003; Carline *et al*., 2005). Therefore, trees were not included in the ecosystem C inventory and due to their low density and immaturity tree seedlings would not have significantly influenced C estimates in this study. All sampled locations and the total area of each sampled exclosure were recorded at each site (Table 1; Table S1).

Sampling was undertaken between May and July in 2010 and 2011, with each site being sampled on a single day. Prior to sampling an exclosure, two 10 × 10 m areas, one inside and the other outside the exclosure, were marked out and all sheep and deer pellets were counted as an estimate of relative grazing intensity (Gilbert *et al*., 2012) inside and outside the exclosure (Table 1). Before collecting plant and soil samples, the maximum height of vegetation was recorded at three randomly selected areas within each 10 × 10 m area (Barthram, 1986). Faecal pellet density and maximum plant height were not recorded for the three sites sampled in 2011 (Fig. 1). These measures showed that the presence of large herbivores maintained a lower sward height of 36.7 cm compared to 51.2 cm inside exclosures (paired *t*‐test; *t* = 4.39, df = 15, *P *<* *0.001) and that fences excluded herbivores (sheep and deer) effectively; pellet densities averaged 0.1 pellets m^−2^ outside exclosures compared to 0.0005 pellets m^−2^ inside exclosures (≈1 pellet recorded in one exclosure) (generalized linear model; *χ* ^2^ = 137.8, df = 1,30, *P *<* *0.001; Table S1). Both vegetation height and pellets were explored as variables explaining plant and soil C stocks.

Plant and soil samples were collected at random coordinates within each 10 × 10 m sampling area. To determine plant C stocks, live aboveground plant material was destructively sampled within a 0.5 × 0.5 m area. Due to high densities of litter within exclosures at some sites, a smaller 0.1 × 0.1 m area of litter (within the live aboveground plant sample area) was collected down to the soil surface. As some exclosures were small <100 m^2^ (Table S1) or part of restoration projects, multiple vegetation samples were not collected. Belowground C stocks comprising combined soil and roots to a depth of 15 cm (hereafter referred to as soil C) were determined from 3 replicate soil cores collected directly below the sampled vegetation using a 4.2‐cm‐diameter corer. Depths of soil horizons were measured *in situ* prior to the soil samples being taken, while moisture content was determined gravimetrically by drying at 80 °C. All vegetation samples and soil cores were kept in an ice‐filled cool box and then stored at 4 °C prior to sorting, typically within 3 days.

Aboveground plant material was separated into the following functional groups: dwarf shrubs (woody species: e.g. *C. vulgaris*,* J. communis* subsp*. nana* and *Erica tetralix*), graminoids (predominately Poaceae with some Cyperaceae), mosses (bryophyte species: e.g. *Hylocomium splendens* and *Hypnum jutlandicum*), forbs (dicotyledonous herbaceous species), ferns and lichens (combined) and plant litter. Ferns and lichens were only found at 4 of 19 sites and accounted for <1% of the plant community biomass on average and were therefore omitted from further data analysis. All aboveground biomass was oven‐dried for 48 h at 80 °C and weighed (±0.01 g). Soil cores were separated into fermentation (plant fibres visible but starting to break down), organic (the remaining organic and humus horizon with organic structures becoming indiscernible) and mineral (low organic matter content) horizons. Samples from each horizon were weighed wet, oven‐dried for 48 h at 105 °C and reweighed dry (±0.01 g) to determine volumetric soil water content (g H_2_O cm^−3^ dry soil). At each site, replicate soil samples from each horizon were pooled within exclosure and within grazed area for chemical analysis. Each plant functional group present inside and outside exclosures at each site was analysed separately. The N and C contents of plant functional groups and soil horizons were determined by homogenizing samples with a steel ball mill (Retsch GmbH, Haan, Germany; Smith *et al*., 2013) to generate a standard 5 mg subsample for elemental analysis (Carlo‐Erba NA 1500 Series 2, USA).

Aboveground plant C stocks (kg C m^−2^) were determined by multiplying plant biomass by its C concentration (%) divided by the sampled area. Soil C stocks represent the mean of three replicate cores; however, 21 of 114 soil cores were <15 cm in depth due to indurated mineral horizons or poor cohesion of soils with high moisture contents. For these cores, soil depth and bulk density of the lower horizon within the core were extrapolated to 15 cm and estimated C stocks adjusted accordingly. A volume‐based measure of soil C stocks (to a depth of 15 cm) was calculated from soil bulk density (without stones >1 mm), core volume and carbon concentration and scaled to kg C m^−2^.

### Climate and N deposition data

To determine the potential effect of climatic conditions on plant and soil C stocks and investigate climate covariation with rates of N deposition and duration of herbivore exclusion, long‐term gridded climate data (5 × 5 km) were obtained for each site from Met Office UKCP09 databases (available via www.metoffice.gov.uk). The spatial resolution of climatic data at 5 × 5 km was the same as total atmospheric N deposition (CBED modelled N data). Long‐term climatic data (1961–2006) were used because they are significant predictors of plant productivity and microbial composition and activity, and therefore likely influence plant and soil C stocks (Prentice *et al*., 2011; De Vries *et al*., 2012). Climatic variables included mean growing season length (period after 1st July when daily mean temperature >5 °C for more than five consecutive days); growing degree days (the day‐by‐day sum of the mean number of degrees by which air temperature is more than 5.5 °C); and average annual rainfall (1981–2010) and values for each site are in Table S1.

### Statistical analysis

The effect of herbivore exclusion, exclosure age, N deposition rate and climatic variables on plant and soil C stocks and N concentrations in plant shoots and litter was explored using linear mixed‐effect models with residual maximum likelihood estimations (REML) in R, lmer package (version 2.10.1, R Development Core Team, 2009; Bates & Maechler, 2010). Multiple fixed variables were explored in all models using the following sequence: exclosure treatment, exclosure age (modelled by the interaction term exclosure treatment × exclosure age), N deposition and climatic variables (growing season length, degree days and mean annual rainfall) and the interaction between exclosure treatment and N deposition. The total variance explained was estimated from the *R* ^2^ of the relationship between the actual data and model‐predicted values and is a measure of goodness of fit for mixed models (De Vries *et al*., 2012). In addition, we used separate linear mixed‐effect models to correlate litter C against the C stocks in the various functional groups of plants using the covariance structure in the model. There was no significant relationship between N deposition and rainfall. The random structure was defined as site to account for the paired sampling design (inside and outside exclosures at each site). The final models were simplified following Akaike's Information Criterion (AIC) and only retained factors found to be significant in chi‐squared likelihood ratio deletion tests (LRTs) (Pinheiro & Bates, 2000). Once the final model was reached, the significance of each term was assessed by removing it from the simplified model and performing LRTs. To obtain goodness of fit for our mixed models, we calculated the *R* ^2^ of the linear regression between the actual data and model‐predicted values (De Vries *et al*., 2012). The plant functional group ‘forbs’ was a minor component of total plant C stocks (averaged ~1% of total plant C stocks) and was included in the total plant C analysis but was not analysed statistically as an individual functional group. All means are presented with standard errors (mean ± SE).

### Estimating heathland C storage across the UK

We determined the combined effects of herbivore removal and current rates of N deposition on potential C storage for all UK heathlands defined here as dwarf shrub communities, dominated by *C. vulgaris* and other ericaceous species with a peat depth <0.5 m (Carey *et al*., 2008; Emmett *et al*., 2010). We combined the area of UK heathland (land cover map from Countryside Survey 2007 using 1 km^2^ grids as a basis; Morton *et al*., 2011) and total atmospheric N deposition (CBED modelled N data) in a geographic information system (GIS) package (ESRI^®^ ArcGISTM 9.3). Individual patches of heathland in the UK (Morton *et al*., 2011) were assigned an average total N deposition rate derived from CBED modelled values within a 5 km radius of each heathland patch. Only heathland areas within the N deposition range of this study (5–24 kg N ha^−1^ yr^−1^) were used, comprising 1.81 million ha which is 94.7% of total heathland area in the UK (Fig. S1 ). We subtracted the ecosystem (i.e. the sum of soil and plant C stocks) and total soil C stocks inside exclosures from outside exclosures at each site across the N deposition gradient, and generated a linear equation describing the relationship between the difference (either negative or positive) in C stock (t ha^−1^) between grazed systems and those from which large herbivores had been removed, and N deposition rate. We used this equation to derive the difference in C stock for each heathland patch across the UK, according to the N deposition rate received by that patch. For each heathland patch, the predicted difference in C stocks as a consequence of grazing removal was multiplied by the land area of the patch. Finally, we summed these values to generate single national values that quantified the net effect of removing large herbivores on both ecosystem and total soil C storage. To compare the effect of including N deposition against excluding it, we repeated the process of upscaling but ignored N deposition effects by applying the overall mean differences in C storage between grazed and exclosed ecosystem and total soil C storage to all the UK heathland patches.

## Results

### Effects of exclusion of large herbivores on plant and soil C stocks

**Figure 2:**
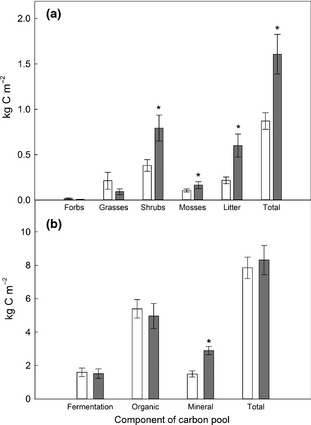
Aboveground plant (a) and soil (b) carbon stocks in paired grazed (open) and exclosed (grey) habitats (±SEM). Asterisks indicate significant difference between grazed and exclosed habitats (*P *<* *0.05; Table 2). Note difference in the scales of the *Y* axes.

Excluding large herbivores significantly increased aboveground plant C storage from 0.87 ± 0.09 kg C m^−2^ in grazed plant communities to 1.61 ± 0.22 kg C m^−2^ inside exclosures. The greater amount of litter, shrub and moss C stocks contributed to the total increase in plant C stocks in exclosures, while C stocks in grasses were not significantly affected by herbivore exclusion (Fig. 2; Table 2). On average, shrub C stocks were 55.8% greater within exclosures compared to grazed communities, litter C stocks were 52% greater and moss C stocks were 8.1% greater while there was a nonsignificant reduction of −17.3% in C stocks in grasses (Fig. 2) in response to exclusion of grazing. Shrub C stocks were the only plant functional group correlated (positively) with litter C stocks (mixed‐effect model; *X* ^2^(1) = 24.58, *P* < 0.001), explaining 62.6% of the variation in both grazed and ungrazed plant communities. Therefore, litter C stocks are likely to be derived primarily from shrubs. The effect of exclosures on plant C stocks was due to an accumulation of plant biomass and litter (Table 2): the C concentrations of plant functional groups were unaffected by exclosures (data not shown). Neither total plant nor functional group C stocks increased with duration of herbivore exclusion (Table 2). In fact, the only observed effect of exclosure age was a decrease in grass tissue N concentration with increasing years of herbivore exclusion (Table 3).

**Table 2:** Effect of grazing exclosures, N deposition rate, exclosure age and mean annual rainfall on aboveground plant carbon (C) stocks (total, litter, shrub, grass and moss), soil C stocks (total, fermentation, organic and mineral horizons; including roots) and ecosystem C stocks (plant and soil combined)

Plant	Total C			Total biomass			Litter C			Shrub C			Grass C			Moss C		
	*χ* ^2^	df	*P*	*χ* ^2^	df	*P*	*χ* ^2^	df	*P*	*χ* ^2^	df	*P*	*χ* ^2^	df	*P*	*χ* ^2^	df	*P*
Exclosure	10.43	1	0.001	10.67	1	0.001	9.17	1	0.002	8.28	1	0.004	_–_	_–_	_–_	10.11	1	0.001
Exclosure age	_–_	_–_	_–_	_–_	_–_	_–_	_–_	_–_	_–_	_–_	_–_	_–_	_–_	_–_	_–_	_–_	_–_	_–_
N deposition	_–_	_–_	_–_	_–_	_–_	_–_	_–_	_–_	_–_	_–_	_–_	_–_	_–_	_–_	_–_	11.53	1	0.007
N deposition × exclosure	_–_	_–_	_–_	_–_	_–_	_–_	_–_	_–_	_–_	_–_	_–_	_–_	_–_	_–_	_–_	7.48	1	0.006
Total rainfall	6.12	1	0.013	6.51	1	0.011	5.57	1	0.018	3.57	1	0.059	13.08	1	<0.001	_–_	_–_	_–_
Total variance explained (%)	33.13			40.4			30.47			65.89			54.22			80.77		

Soil C	Total			Fermentation			Organic			Mineral			Ecosystem C		
	*χ* ^2^	df	*P*	*χ* ^2^	df	*P*	*χ* ^2^	df	*P*	*χ* ^2^	df	*P*	*χ* ^2^	df	*P*
Exclosure	3.87	1	0.049	_–_	_–_	_–_	_–_	_–_	_–_	8.14	1	0.004	0.97	1	0.32
Exclosure age	_–_	_–_	_–_	_–_	_–_	_–_	_–_	_–_	_–_	_–_	_–_	_–_	_–_	_–_	_–_
N deposition	17.87	1	<0.001	3.65	1	0.056	11.13	1	<0.001	_–_	_–_	_–_	14.21	1	<0.001
N deposition × exclosure	6.11	1	0.013	_–_	_–_	_–_	_–_	_–_	_–_	_–_	_–_	_–_	3.78	1	0.052
Total rainfall	_–_	_–_	_–_	_–_	_–_	_–_	_–_	_–_	_–_	_–_	_–_	_–_	3.14	1	0.076
Total variance explained (%)	94.37			75.9			83.9			50.58			91.85		

Models have been simplified to retain significant terms following likelihood ratio deletion tests (LRTs). The total variance explained is a measure of goodness of fit for mixed models, estimated from the *R* ^2^ of the relationship between the actual data and model‐predicted values (De Vries *et al*., 2012). For each factor, chi‐square values (*X* ^2^), associated degrees of freedom (df) and *P*‐values describe the effect of removing a factor from the final simplified model. Dashes indicate factors that were removed from the initial model.

**Table 3:** Effect of grazing exclosures and N deposition rate on N concentrations (%) in plant functional groups (litter, shrub, grass and moss) and soil horizons (fermentation, organic and mineral)

Plant % N	Litter			Shrub			Grass			Moss		
	*χ* ^2^	df	*P*	*χ* ^2^	df	*P*	*χ* ^2^	df	*P*	*χ* ^2^	df	*P*
Exclosure	_–_	_–_	_–_	_–_	_–_	_–_	3.96	1	0.047	_–_	_–_	_–_
Exclosure age	_–_	_–_	_–_	_–_	_–_	_–_	8.07	1	0.005	_–_	_–_	_–_
N deposition	11.19	1	<0.001	_–_	_–_	_–_	_–_	_–_	_–_	6.74	1	0.013
N deposition × exclosure	_–_	_–_	_–_	_–_	_–_	_–_	_–_	_–_	_–_	_–_	_–_	_–_
Total rainfall	_–_	_–_	_–_	2.40	1	0.121	7.69	1	0.006	_–_	_–_	_–_
%N	1.41 ± 0.06	0.97 ± 0.06	1.30 ± 0.04	1.22 ± 0.06								
C:N ratio	32.54 ± 0.24	54.06 ± 3.26	36.02 ± 1.10	40.43 ± 1.82								
Soil N	Fermentation	Organic	Mineral									
%N	1.45 ± 0.09	1.34 ± 0.11	0.40 ± 0.07									
C : N ratio	23.67 ± 1.05	20.91 ± 0.09	20.90 ± 1.34									

Models have been simplified retaining significant terms following likelihood ratio deletion test (LRTs). For each factor, chi‐square values (*X* ^2^), associated degrees of freedom (df) and *P*‐values describe the effect of removing a factor from the final simplified model. Plant and soil N concentrations (%) and C : N quotients (means ± 1 SE) are shown for the average of ungrazed and grazed habitats across all sites. Dashes indicate factors that were removed from the initial model.

In contrast to the differences observed in total aboveground plant C, excluding large herbivores had little overall effect on total soil C storage (soil plus roots, to 15 cm depth), which averaged 8.32 ± 0.87 kg C m^−2^ inside exclosures and 7.85 ± 0.64 kg C m^−2^ under grazed communities across all sites (Fig. 2). Although this small effect was statistically significant, actual differences in soil C between grazed and ungrazed vegetation depended far more strongly on N deposition, as described below. Total aboveground plant C was not correlated with total soil C or any individual soil horizon C pool (data not shown). Total and individual soil horizon C and N concentrations, bulk density and soil moisture content did not significantly differ between exclosures and grazed areas (data not shown). There was an apparent difference in the distribution of soil C stocks within the soil profile; a greater proportion of the total soil C stock was found within the mineral horizon inside exclosures compared to adjacent grazed areas (Fig. 2; Table 2). This was because the depth of the overlying fermentation and organic horizons inside exclosures was reduced by 8% on average compared to grazed areas (Fig. S2; mixed‐effect model; *X* ^*2*^ **=* *5.93, df = 1, *P *=* *0.015), resulting in the inclusion of a greater depth of mineral soil at the bottom of these cores. Importantly, this reduction in upper soil horizon depth in exclosures did not alter total soil C storage, primarily due to the large variability within the organic horizons in exclosures (4.97 ± 0.75 kg C m^−2^; mean ± SE) and under grazed communities (5.39 ± 0.55 kg C m^−2^). Older exclosures did not accrue more soil C, even given the wide range of exclosure ages including many several decades old (Table 1).

**Figure 3:**
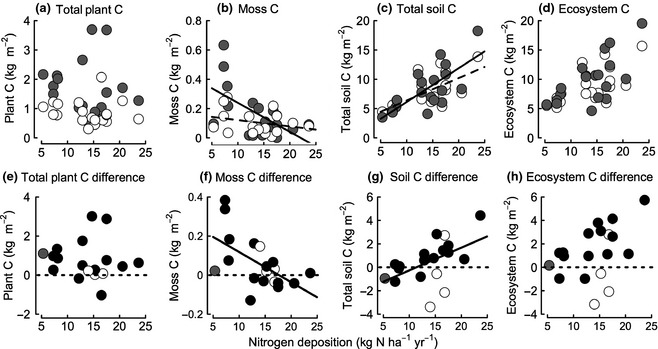
Total aboveground plant (a), moss (b), total soil (c) and ecosystem (combined plant and soil) (d) carbon stocks in paired grazed (white symbols) and exclosed (grey symbols) habitats in relation to N deposition; linear model fits for grazed communities are shown with a dashed line and exclosures with a solid line. The difference in total aboveground plant (e), moss (f), total soil (g) and ecosystem (h) carbon stocks, between grazed and exclosed habitats in relation to N deposition. The solid line is the fitted linear relationship for all sites, and symbols represent dominant exclosure vegetation types; *Calluna vulgaris* in black, *Molinia caerulea* in white and *Juniperus communis* subsp*. nana* in grey. The dotted line represents no difference between grazed and exclosed plant and soil carbon stocks.

Overall, removing herbivores increased ecosystem C storage to 10.01 ± 0.96 kg C m^−2^ compared to adjacent grazed areas 8.74 ± 0.68 kg C m^−2^ (Fig. 3d,h), an effect which was driven by an interaction with N deposition. Positive effects of herbivore removal on C stocks were generally greater for exclosures dominated by shrub species *C. vulgaris* and *J. communis* subsp.* nana* (>40% of the live plant community biomass) than by the grass *M. caerulea* at sites receiving similar rates of N deposition (Fig. 3g,h; Table 1). However, as only four sites had exclosures dominated by *M. caerulea,* we were unable to explore this relationship statistically.

### Direct effects of atmospheric N deposition on C stocks

Nitrogen deposition correlated strongly and positively with shrub (combined leaf and stem) C concentrations (mixed‐effect model; *X* ^*2*^ **=* *10.93, df = 1, *P *<* *0.001), but this did not result in a positive effect of N deposition on aboveground shrub C stocks (Table 2). Indeed, total aboveground plant C, either inside or outside exclosures, was not associated with N deposition rate (Fig. 3a). The only plant functional group C stock associated with N deposition was in moss, with C stocks declining with increasing N deposition in both grazed and ungrazed plant communities (Fig. 3b). However, moss comprised only 14.0 ± 1.9% of the total plant C pool averaged for inside and outside exclosures.

Nitrogen deposition also influenced N concentrations in plant litter and moss, which had increases of 0.049% and 0.034% N per kg ha^−1^ year^−1^ of deposited N, respectively, but there was no significant effect on shrub or graminoid tissue chemistry (Table 3).

Total soil C storage increased significantly with increasing atmospheric N deposition (Table 2; Fig. 3c) by about 0.45 kg C for every 1 kg N, with increases only in the organic layer, although there was a trend for an increase in the fermentation horizon (*P *=* *0.056; Table 2). Soil horizon depths, water content and C and N concentrations were not significantly correlated with increasing N deposition, but C to N ratios followed the same correlation as total soil C stocks (data not shown).

### Influence of N deposition on C stock response to herbivore exclusion

There was a significant interaction between the rate of N deposition and herbivore removal both on soil and ecosystem C stocks (Table 2). Removing herbivores resulted in an increase in soil C stocks for heathland sites receiving more than ~11 kg N ha^−1^ year^−1^, while below this N deposition, threshold removing herbivores resulted in a marginal reduction in soil C stocks (Table 2; Fig. 3g). For the ecosystem (plants + soil), the threshold above which herbivore removal resulted in increased C stocks was lower, at ~7 kg N ha^−1^ year^−1^ (Table 2; Fig. 3h). The same combined effects of N deposition and herbivore activity were not observed for total plant C stocks. However, the greatest change in moss C stocks within exclosures occurred at low rates of N deposition, and there was an apparent threshold of 17 kg N ha^−1 ^year^−1^ above which there was little difference between grazed and ungrazed moss C (Fig. 3f).

### What impact would herbivore removal from all UK heathlands have on C stocks given current spatial distribution and rates of N deposition?

**Figure 4:**
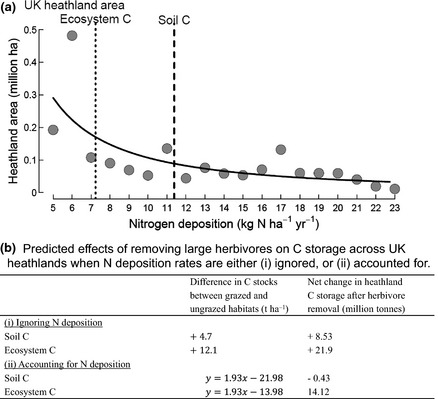
(a) The area of heathland (plant communities dominated by the dwarf shrub *Calluna vulgaris* covering 1.9 million ha*;* Emmett *et al*., 2010) in the UK categorized into 1 kg N ha^−1^ year^−1^ increments of N deposition (i.e. first symbol is 5–6 kg N ha^−1^ yr^−1^) in the range 5–24 kg N ha^−1^ year^−1^(Smith *et al*., 2000; Morton *et al*., 2011). The solid line is the fitted nonlinear relationship for heathland area within each kg N deposition category across the N deposition gradient. The dashed line represents the threshold (~11 kg N ha^−1^ yr^−1^) above which soil C inside exclosures exceeds that outside exclosures, the dotted line represents the equivalent threshold (~7 kg N ha^−1^ yr^−1^) for ecosystem C (i.e. total plant and soil C stocks). (b) The predicted effect of removing herbivores on soil and ecosystem C storage for the total area of UK heathlands using the difference in soil and ecosystem C inside and outside exclosures when N deposition is (1) ignored or (2) accounted for. In (2), *y* is the difference in C stocks between grazed and exclosed vegetation and *x* is the N deposition rate for each patch of heathland. For each patch, the predicted difference in C stocks as a consequence of grazing removal was multiplied by the land area of the patch, and these values were summed to generate single national values that quantify the net effect of removing large herbivores.

Scaling‐up the average differences in ecosystem and soil C storage following herbivore removal to the total land area of UK heathlands (ignoring N deposition) results in predicted increases in ecosystem C storage of 21.9 million t C and in soil C storage of 8.5 million t C (Fig. 4). Carbon storage was dependent on the combined effects of herbivore exclusion and N deposition, with herbivore removal only resulting in increased C storage where N deposition exceeded a threshold. The threshold for ecosystem C storage (7 kg N ha^−1 ^yr^−1^) was below that for soil C storage (~11 kg N ha^−1 ^yr^−1^; Fig. 3d,h; Table 2). Much of UK heathland is in areas of relatively low N deposition; we estimate that 61.9% of UK heathlands receive sufficient atmospheric N for herbivore removal to result in an increase in net ecosystem C storage (> kg N ha^−1^ yr^−1^; Fig. 4). Moreover, only 41.7% of the UK heathlands occur above the N deposition threshold that would result in a gain in soil C following herbivore removal (~11 kg N ha^−1 ^yr^−1^; Fig. 4). Strikingly, scaling‐up combined effects of N deposition rates and herbivore removal indicates that UK heathland ecosystem C storage would increase by only 14.1 million t C, which is 35% less than when only herbivore removal was considered (Fig. 4). Moreover, UK heathlands would also be expected to lose 0.43 million t C from the soil, rather than gain C in soil, as removal of herbivory results in marginally negative effects on soil C in areas of low N deposition (Fig. 4).

## Discussion

Surveying long‐term (up to eighty years old) exclosures across the UK uplands has demonstrated that the removal of large herbivores from *C. vulgaris‐*dominated wet upland heathlands will increase aboveground plant C storage. However, an increase in soil C storage, which is 5–10 times greater than aboveground plant C storage (Fig. 1), following herbivore removal depends on atmospheric N deposition, and only occurs at higher deposition rates (~11 kg N ha^−1^ yr^−1^). The mechanisms behind the response to this deposition rate are unclear, but the threshold may reflect a crucial change in soil microbial activity or chemistry that ultimately affects C storage. In the nutrient‐limited systems we studied, the positive effects of N deposition on ecosystem C storage outweighed the effect of herbivore removal and exclosure duration. Our results suggest that the combined effects of herbivore removal and regional variation in N deposition need to be given greater recognition. On a national scale, we found that ignoring the effects of N deposition led to considerable overestimates of C storage following herbivore removal because most heathlands are found in areas of low N deposition. We recognize, however, that our scaling exercise did not consider the influence of historical management practices and grazing density and within‐community heterogeneity.

Numerous studies have argued that the presence of herbivores either accelerates or decelerates N cycling within an ecosystem depending on their influence on plant species composition and hence the quantity and quality of litter production, which affects accumulation of soil C (Pastor & Cohen, 1997; Frank & Groffman, 1998; Ritchie *et al*., 1998). The decline in grass tissue N concentration with exclosure duration suggests that herbivore removal slows N cycling in our study system, and this may explain the small response of ecosystem C stocks to herbivore removal in areas of low N deposition. Overall, herbivore removal increased plant biomass and C stocks, most notably the C stocks in shrubs and hence in litter, which was primarily derived from the dominant dwarf shrub *C. vulgaris*. In *C. vulgaris*‐dominated communities, fertilization with N has been shown to increase herbivore off‐take (Emmett *et al*., 2004), reducing plant litter C inputs to the soil. However, there was no such interactive effect of N deposition and herbivory on litter C stock in our study, neither was there any strong correlation between N deposition and litter and total plant or total soil C stocks. The increase in soil C following herbivore removal at high rates of N deposition could not, therefore, be attributed to an increase in aboveground plant biomass, a situation seen in other studies on grazed heathlands (Ward *et al*., 2007; Medina‐Roldán *et al*., 2012; Quin *et al*., 2014).

There was no evidence of N deposition altering C pools in aboveground litter, which was primarily derived from shrubs, and plant biomass, although the amount of C held in moss tissue was negatively associated with N deposition. It is possible that N deposition could have increased the quantity of shrub root C input to the soil (Liu & Greaver, 2010). The proportion of root C that is recalcitrant is greater in *Calluna* than in grasses (Quin *et al*., 2014), and in grazed upland communities, decomposition of root litter is strongly influenced by plant species composition and their associated traits (Smith *et al*., 2014b). Our finding that N deposition affected total soil C storage in the organic horizon, where most roots are located, indicate changes in root litter or rhizodeposition may contribute to soil C pools.

Removing large herbivores alters the abundance of plant functional groups, and in this study, both moss and shrub C stocks increased, as with similar changes observed in other northern ecosystems (Hartley & Mitchell, 2005; Olofsson *et al*., 2009; Armitage *et al*., 2011). Yet in our study, moss C stocks declined with increasing N deposition eventually resulting in little difference inside and outside exclosures. Mosses can govern the rate of C accrual due to their recalcitrant litter and effects on microclimatic controls of decomposition (Gornall *et al*., 2007; Woodin *et al*., 2009), and declines in moss abundance with increasing N deposition have been negatively correlated with ecosystem C storage (Bragazza *et al*., 2012; Larmola *et al*., 2013). However, mosses were a minor component of the plant communities we sampled and probably had a minor effect on soil C pools.

The removal of herbivory resulted in greater increases in ecosystem C stocks in dwarf shrub‐dominated areas compared to graminoid (*M. caerulea*)*‐*dominated communities receiving similar rates of N deposition (Fig. 3h). *M. caerulea* is a tussock‐forming grass with shoot bases that are dense stores of C (Smith *et al*., 2014a), but its roots decompose more quickly than co‐occurring graminoids (Smith *et al*., 2014b) and its tissues contain a lower proportion of recalcitrant C than in *C. vulgaris* (Quin *et al*., 2014). Thus, *M. caerulea* decomposes more readily than *C. vulgaris*, which may contribute to the apparent lack of effect of herbivore removal on C stocks in *M. caerulea*‐dominated habitats. On a national scale, long‐term declines in *C. vulgaris* and replacement by *M. caerulea* across UK upland heathlands (Ross *et al*., 2012) are likely to reduce the impact of herbivore removal on net ecosystem C storage.

Large herbivore grazing has been identified as a potential management tool to influence ecosystem C storage, yet synthesis of studies undertaken across the globe shows the direction of effects to be either positive or negative (Piñeiro *et al*., 2010; Tanentzap & Coomes, 2012; Smith *et al*., 2014a). This has been attributed to variable lengths of time since removing herbivores; however, in our study, exclosure age did not significantly influence long‐term C storage. Instead, regional variation in atmospheric N deposition is a significant environmental driver that may explain the variable effect of removing herbivores on ecosystem C storage. Here, we scaled‐up our findings to estimate the combined effects of herbivore exclusion and N deposition for UK heathland C storage, most of which is found in Scotland and northern England. However, we also highlight the predictions did not use historical management and grazing intensity, or community‐scale heterogeneity in response to grazing and N deposition, all of which require further research. While acknowledging these limitations, we found that for heathlands with low N deposition rates, removing herbivores will have little impact on C storage, while at high N deposition rates, excluding herbivores will enhance ecosystem C storage. The critical load for ungrazed wet upland heathlands is suggested to be close to 10 kg N ha^−1^ year^−1^ (Bobbink & Hettelingh, 2010) and, for the first time, this study identifies that C accrual resulting from herbivore removal from heathlands at or below this critical load will be limited. On a national scale, a spatially explicit approach is therefore needed for heathland grazing management to enhance C storage, one that recognizes interactive effects with regional variation in atmospheric N deposition.

## Supporting information


**Table S1.** Surveyed long‐term upland exclosure details; location, age, size (m^2^) (calculated from http://digimap.edina.ac.uk/digimap/home), large herbivores excluded by fencing, mean annual rainfall, growing season length and growing season degree days (Met Office UKCP09 databases; http://www.metoffice.gov.uk/climatechange/science/monitoring/ukcp09/).
**Figure S1.** Total UK heathland area integrated with atmospheric N deposition from 2011 (area defined as ‘heath’ class in the Countryside Survey 2007 landcover map; Morton *et al*., 2011).
**Figure S2.** Depths of each soil horizon (from soil surface at 0 cm) under grazed and exclosed heathland communities, which were sampled to a maximum depth of 15 cm.
