# Supplementary material for: Combination of herbivore removal and nitrogen deposition increases upland carbon storage
Source: Glob Chang Biol. 2015 Apr 30;21(8):3036–48. doi: 10.1111/gcb.12902 (PMC4973882; doi:10.1111/gcb.12902)
Supplement: Supplementary file 1 — Table S1. Surveyed long‐term upland exclosure details; location, age, size (m2) (calculated from http://digimap.edina.ac.uk/digimap/home), large herbivores excluded by fencing, mean annual rainfall, growing season length and growing season degree days (Met Office UKCP09 databases; http://www.metoffice.gov.uk/climatechange/science/monitoring/ukcp09/). Figure S1. Total UK heathland area integrated with atmospheric N deposition from 2011 (area defined as ‘heath’ class in the Countryside Survey 2007 landcover map; Morton et al., 2011). Figure S2. Depths of each soil horizon (from soil surface at 0 cm) under grazed and exclosed heathland communities, which were sampled to a maximum depth of 15 cm. [file GCB-21-3036-s001.docx]

## SUPPLEMENTARY INFORMATION

**Table S1** Surveyed long-term upland exclosure details; location, age, size (m^2^) (calculated from <http://digimap.edina.ac.uk/digimap/home>), large herbivores excluded by fencing, mean annual rainfall, growing season length and growing season degree days (Met Office UKCP09 databases; <http://www.metoffice.gov.uk/climatechange/science/monitoring/ukcp09/>).

| Site | National grid reference | Size  (m^2^) | Herbivores excluded | Mean rainfall 1981-2010 (mm y^-1^) | Growing season | |
| --- | --- | --- | --- | --- | --- | --- |
|  |  |  |  |  | Length | Degree days |
| Ballogie | NO557935 | 851 | Sheep & deer | 825 | 262.53 | 1250.3 |
| Beinn Eighe | NG980626 | 3747 | Deer | 2596 | 317.87 | 1027.34 |
| Ben Lawers | NN611381 | 247851 | Sheep & deer | 2305 | 189.8 | 756.3 |
| Bowland | SD625502 | 99387 | Sheep | 1528 | 242.53 | 1119.88 |
| Creag Meagaidh  (plot C) | NN463867 | 100 | Deer | 2083 | 218.67 | 833.5 |
| Creag Meagaidh  (plot D) | NN455859 | 100 | Deer | 2083 | 218.67 | 833.5 |
| Crianlarich | NN350301 | 28000 | Sheep | 3169 | 234.43 | 972.49 |
| Geltsdale | NY645580 | 42363 | Sheep | 951 | 273.00 | 1326.92 |
| Glen Clunie | NO139820 | 24 | Sheep, deer & hares | 1177 | 200.27 | 814.41 |
| Glen Finglas  (block B) | NN529109 | 32770 | Sheep & cattle | 2595 | 175.53 | 617.1 |
| Glen Finglas  (block C) | NN483122 | 32300 | Sheep & cattle | 2600 | 268.07 | 1226.4 |
| Glen Finglas  (block E) | NN515141 | 32923 | Sheep & cattle | 2595 | 175.53 | 617.1 |
| Glen Loy | NN093837 | 369053 | Sheep & deer | 3028 | 224.23 | 807.59 |
| Glen Shee | NO125725 | 24 | Sheep, deer & hares | 1636 | 140.07 | 406.27 |
| Glensaugh  (MOORCO) | NO675799 | 2352 | Sheep & deer | 1239 | 217.30 | 877.16 |
| Glensaugh (Strathfinella Hill) | NO677780 | 1900 | Sheep & deer | 1222 | 215.57 | 867.73 |
| Invercauld | NO165946 | 750 | Sheep & deer | 1284 | 152.37 | 499.66 |
| Invernaver | NC694616 | 121 | Sheep & hares | 1066 | 304.63 | 1249.93 |
| Loch na Lairgie | NN593412 | 565588 | Sheep | 9.09 | 178.17 | 653.61 |


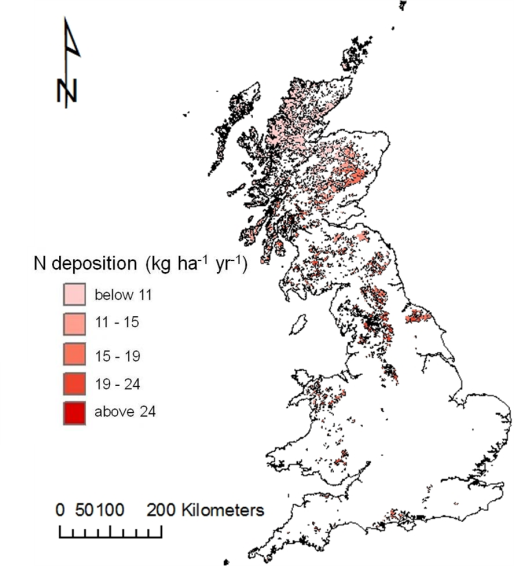


**Figure S1.** Total UK heathland area integrated with atmospheric N deposition from 2011 (area defined as ‘heath’ class in the Countryside Survey 2007 landcover map; Morton *et al.,* 2011). Only heathlands receiving N deposition within the range 5 - 24 kg N ha^-1^ yr^-1^ are mapped. (<http://pollutantdeposition.defra.gov.uk/pollutant-map>; Smith *et al.* 2000).

**Figure S2.** Depths of each soil horizon (from soil surface at 0 cm) under grazed and exclosed heathland communities, which were sampled to a maximum depth of 15cm. If the mineral horizon was not detected to a depth of 15 cm it was assumed to have a depth of zero. Soil horizons are means of 19 sites (means ± 1 SE).
